# Volumetric assessment and longitudinal changes of subcortical structures in formalinized Beagle brains

**DOI:** 10.1371/journal.pone.0261484

**Published:** 2022-10-07

**Authors:** Francesca Del Signore, Germain Arribarat, Leonardo Della Salda, Giovanni Mogicato, Alexandra Deviers, Benjamin Cartiaux, Massimo Vignoli, Patrice Peran, Francesco de Pasquale

**Affiliations:** 1 Veterinary Faculty, University of Teramo, Teramo, Italy; 2 ToNIC Toulouse Neuroimaging Center UMR1214—Inserm/UPS, Tolouse, France; University of Minnesota, UNITED STATES

## Abstract

High field MRI is an advanced technique for diagnostic and research purposes on animal models, such as the Beagle dog. In this context, studies on neuroscience applications, e.g. aging and neuro-pathologies, are currently increasing. This led to a need for reference values, in terms of volumetric assessment, for the structures typically involved. Nowadays, several canine brain MRI atlases have been provided. However, no reports are available regarding the measurements’ reproducibility and little is known about the effect of formalin on MRI segmentation. Here, we assessed the segmentation variability of selected structures among operators (two operators segmented the same data) in a sample of 11 Beagle dogs. Then, we analyzed, for one Beagle dog, the longitudinal volumetric changes of these structures. We considered four conditions: *in vivo*, *post mortem* (after euthanasia), *ex vivo* (brain extracted and studied after 1 month in formalin, and after 12 months). The MRI data were collected with a 3 T scanner. Our findings suggest that the segmentation procedure was overall reproducible since only slight statistical differences were detected. In the *post mortem/ ex vivo* comparison, most structures showed a higher contrast, thereby leading to greater reproducibility between operators. We observed a net increase in the volume of the studied structures. This could be justified by the intrinsic relaxation time changes observed because of the formalin fixation. This led to an improvement in brain structure visualization and segmentation. To conclude, MRI-based segmentation seems to be a useful and accurate tool that allows longitudinal studies on formalin-fixed brains.

## Introduction

High-field MRI, characterized by high spatial resolution, high SNR, and short acquisition time, represents an advanced technique to investigate animal models, such as the dog [1,2]. This model offers several advantages over rodent and primate ones, as testified by the growing literature on neurocognitive, aging, and clinical applications [3,4]. Neurocognitively, the canine shares similar behavioral/emotional responses with humans, e.g. in linking learning, memory, and other cognitive functions. These convergent sociocognitive skills place the dog in a unique position to increase our understanding of these aspects in humans [5]. Among domestic canines, the Beagle is the breed most commonly used in laboratories, thanks to its moderate size, docile nature, and strong immunity [6–8]. The dog brain, being gyrencephalic, as compared to rodent and avian, represents a better experimental model for several disorders, e.g. gliomas and aging [9–11]. In this context, studies typically focus on the volumetric assessment of specific brain structures, thus the need for reference values for them. To provide a common spatial referencing and architectonic-based cortical segmentation, recent studies developed a standard atlas for coregistered data [5]. Similarly, a stereotactic cortical atlas for the mesaticephalic canine brain has been developed for functional and structural MRI analyses [3]. However, the currently available atlases, see for example [12–14], are affected by some limitations, e.g. a small sample size [12], the acquisition of non-isovolumetric data [14], the use of dogs non neurologically/clinically healthy [13] and samples composed by mixed breeds [3]. To overcome the uncertainty related to the breed variability, very recently Liu et al. realized a specific atlas for the Beagle breed [15]. All these studies were performed on alive subjects, apart from [12], where formalin-fixed brains *(ex vivo*) were segmented. They provided a diffeomorphic brain atlas of mesaticephalic dogs coregistered onto an *in vivo* template [12]. In this study, some important aspects such as the reproducibility of the measurements across different operators and the volumetric variation from *in vivo* to *ex vivo* phases were not assessed [12]. These aspects are very important, since MRI findings can be linked and often validated through histopathology. This can be very time-consuming and challenging for several reasons, e.g. the inaccurate correspondence of MRI-anatomical sections (due to different slice thickness and orientations) [16]. When whole-brain histopathology is not feasible, *in vivo* and *post-mortem* MRI can be used as a guide for limited pathological sampling [17,18]. Similarly, in forensic radiology, *post-mortem* MRI has been recognized as a supplementary tool to address specific forensic questions [19,20]. However, *ex-vivo* MRI can be very challenging. First, after death, the brain undergoes several changes: microbial degradation, autolysis, breakdown of cell membranes, and stochastic diffusion of molecules. Second, chemical fixation, needed to ensure longitudinal stability of macromolecular structures, also affects tissue properties. Thus, due to death and fixation, a series of artifacts and changes in tissue properties are expected. This will affect MR signals and the conclusions based on MRI measurements in fixed tissue may not directly reflect the *in-vivo* environment [20]. For example, it has been reported that formalin fixation causes a temporally dependent tissue shrinkage that might be inhomogeneous among the various brain structures [21]. However, these aspects are still under debate and the literature is scarce. For this reason, in this study, we analyzed the effect of long-term fixation (12 months) on brain structures in a sample of 11 Beagle dogs. We manually segmented a set of subcortical structures, e.g., Globus Pallidus, Caudate Nucleus, and Substantia Nigra. The regions were chosen since their volumetric changes have been reported to correlate with many neurodegenerative disorders such as Parkinson’s [22]. First, we assessed the variability of the extracted volumes among operators (two operators segmented the same data) and their intrinsic variability within the sample. Then, we analyzed for one further dog the longitudinal changes in the brain segmentation of these structures corresponding to four conditions: *in vivo*, *post mortem* (after euthanasia), *ex vivo* (brain extracted and studied after 1 month in formalin and after 12 months). The last condition overlaps with the previous sample of 11 dogs. As far as we know, this is the first study reporting brain structures in formalinized dogs and their longitudinal changes.

## Materials and methods

### Animals

A sample of 12 healthy Beagle dogs was evaluated in two studies. In the first study, a group of 11 dogs (9 females and 2 males, 1.6 ± 0.2 years) was used to evaluate the effect of long-term fixation on MRI properties of the brain. In this context, a single MRI scan was performed on isolated heads that remained fixed for 11 months. Dogs originated from a laboratory in which they completed their research time and were euthanized for teaching purposes (i.e. preparation of veterinary anatomical teaching materials: MRI brain atlas and embalmed cadavers for dissection).

In the second study, one dog (male, 2 years) was used for the longitudinal evaluation of the effect of death and fixation on MRI. This dog underwent 4 MRI exams: 1 *in vivo*, 1 *post-mortem* performed just after euthanasia, and 2 *ex vivo* performed on the brain removed from the skull. One exam after 1 (*ex_vivo_1*) and 12 months of fixation (*ex_vivo_12*). While the terms *post-mortem* and *ex vivo* are normally interchangeable, in this study, they refer to two different conditions which are the evaluation of the brain confined by the skull just after death (*post-mortem*) and the evaluation of formalin-fixed brains (*ex vivo*, either isolated or confined by the skull). The experimental procedures related to the preparation of veterinary anatomical teaching materials were approved by the Animal Ethics Committee of the National Veterinary School of Toulouse with authorization n° 21559–2019071917392588. Dogs were euthanized by an intravenous (IV) injection of ≥100 mg/kg of sodium pentobarbital while they were deeply anesthetized (anesthetic protocol: IV injection of butorphanol (0,4 mg/kg), medetomidine (20 μg/kg), and diazepam (0,2 mg/kg)). Heparin sodium (1000 IU) was injected by IV route 5 minutes before euthanasia to optimize post-mortem perfusion of fixative solution.

### Fixation protocol

Dogs were anesthetized to acquire in vivo MR images (not used in the present study) after which they were euthanized. Their heads were fixed according to the procedure described above. Heads were then stored in containers filled with 10% formalin solution and were scanned after 11 months of fixation.

For the dog in the second study, in vivo MRI scans and euthanasia were carried out under anesthesia with the protocol described above. *Post-mortem* MRI was performed immediately after euthanasia and once this acquisition was completed, the cadaver was transferred to a special room for fixation. The head was then separated from the body to be perfused via the common carotid arteries with a rinsing solution (NaCl, flow rate: 15 mL/minute, perfusion time: 5 minutes) and a fixative solution (10% formalin solution, 15 mL/minute, perfusion time: 5 minutes). The head was stored in a container filled with 10% formalin solution. After one month of fixation, the brain was removed from the skull for an ex vivo MRI acquisition. An additional ex vivo MRI examination of the brain was performed after 11 months of fixation.

### MRI acquisition

MRI examinations were performed at the Institute for Brain Sciences of Toulouse using a high field 3.0 Tesla magnet (Philips ACHIEVA dStream) at the Inserm/UPS UMR1214 ToNIC Technical Platform. An 8-channel-human elbow coil (serving as dog head coil) was used for signal reception. The *ex-vivo* examinations were performed with a 1-channel solenoid antenna. To guarantee a homogeneous and accurate signal, no acceleration or preparation factors were used. For the longitudinal study, the imaging protocol included T1 and T2 weighted images. The 3D whole-brain T1 and T2 weighted images were acquired in the sagittal plane. For T1 imaging (Fast Field Echo), the sequence parameters were as follows: echo time TE = 4.0 ms, repetition time TR =  9.0 ms, flip angle = 8°. For T2 imaging (Spin Echo) the sequence parameters were the following: TE/TR =  266/2500 ms, flip angle = 90°. The spatial resolution parameters were the same for both acquisitions: pixel spacing 0.5×0.5 mm^2^, slice thickness =  0.5 mm, matrix size = 288×288, numbers of slices = 300, voxel size = 0.5×0.5×0.5 mm^3^, no slice gap. The total duration of the imaging protocol was 60 min. Twenty-four hours before the *ex vivo* scans, the brain was rinsed with water and then submerged in a 0.9% saline solution (NaCl). Just before acquisition, the brain was put in an MRI-compatible container (a plastic container with a leakproof screw cap) filled with saline solution. For the acquisition, they were dried, wrapped up in hermetic packages, held horizontally on the MRI table, and placed in the human elbow coil. The imaging protocol comprised T1-weighted images (TR = 8.5 ms; TE = 3.8 ms; voxel size 0.5×0.5×0.5 mm^3^, matrix 288×288×300) and T2-weighted images (TR = 265.71 ms; TE = 2500 ms; voxel size 0.5×0.5×0.5 mm^3^, matrix 288x288x300).

For the image coregistration, the in vivo T1w MRI data were used as reference volumes for the *ex vivo* data. Then, a rigid transformation was applied (rotation + translation parameters). The adopted similarity function was the Mutual Information with a trilinear resampling method. No scaling was involved in the data transformation.

### Assessment of inter-operator reproducibility

Two veterinarians experienced in canine brain structure, denoted “operators” (O1- and O2 respectively) blindly segmented the following structures: Ventricles (from T1 scans) and Caudate Nucleus, Hippocampus, Substantia Nigra, Putamen, Globus Pallidus, Lateral and Medial Geniculate Nucleus (from T2 scans). They divided the measurements in left and right parts on the group of 11 dogs and for the longitudinal study. The choice to perform the segmentations either in T1 or T2 was driven by the contrast observed in the various structures, i.e. the subjective ease of visualizing specific structures, especially the smaller ones. We did not perform any image transformation to enhance WM/GM contrast, as in [23]. Both the operators considered the contrast enough to perform the segmentation. However, we acknowledge that this aspect could be evaluated in future studies. Here, we adopted a manual segmentation approach, although an automatic segmentation, such as in [14,24], could have been more accurate. Nevertheless, because of the formalin effect on T1/T2 contrast, the latter approach does not seem to perform well with our data (these aspects are elaborated in the next sections). Regarding the analysis on the group of 11 dogs, on each structure, the mean volume (M) and its standard deviation (SD) were evaluated. The volume was provided by ITK SNAP after the segmentation. In these analyses, we did not compute the global brain tissue volume, since we focused on the specific features of single subcortical structures. In a group-level analysis, such a covariate could have been included to account for the brain size across different subjects. However, our results will not be based on a group-level analysis. We studied the effects of each ROI, in each dog, separately. To account for potential size variations, we normalized each structure but its volume.

In fact, to evaluate the percentage of intra-operator reproducibility between different structures, we used the coefficient of variation (CV) defined as CV = SD/M. To assess the agreement of the segmentation performed by the two operators, we computed the Dice similarity indices for each structure [25]. Finally, the statistical comparison between the two operators was provided by a t-test on the mean volumes.

### Longitudinal study

Here, the operators performed the previous segmentations longitudinally. The percentage (%) changes in volume were computed for the phases: *in-vivo*, *post-mortem*, *ex vivo 1 month*, *and ex-vivo 12 months*. Then, volume changes were compared across the operators. We considered the following conditions: *in vivo* vs *post mortem* (defined as (post mortem–in vivo)/in vivo*100), the *post mortem* vs *ex vivo*– 1-month volumes (expressed as (*ex_vivo_1* –*post mortem)/ post mortem* *100), the *in vivo* vs *ex vivo* 1 month volumes (expressed as (*ex_vivo_1* –*in vivo)/ in vivo* *100) and, finally, *ex vivo* 1 month vs *ex vivo* 12 months (expressed as ((*ex_vivo*_12 –*ex_vivo*_1)/ ex_vivo_1*100)).

As in the previous stage, ITK SNAP software (version 3.8.0, 2019) was employed for the manual segmentation and the statistical analyses were performed with MATLAB. The statistical significance was defined through an alpha level of 0.05.

The acquired data are available to the scientific community. They can be downloaded at the public repository https://doi.org/10.6084/m9.figshare.20066351, see Supplementary Information for details.

## Results

### Brain segmentation after 12 months in formalin

The volumes estimated from the segmentations of the two operators after 12 months in formalin are reported in Table 1.

**Table 1:** Summary table for the sample of 11 dogs. Estimated volumes of the segmented structures from operators 1 and 2 (O1/O2), expressed as the volume averaged across the considered subjects, their standard deviation, and CV. For the ventricles, we report the values obtained from the automatic segmentation, since this outperformed the manual approach. The Dice similarity index shows a good agreement between the two operators, corresponding to an average Dice index of 0.75.

**AUTOMATIC SEGMENTATION**						
	**Mean ± St. Dev (mm** ^**3**^ **)**	**CV%**				
**Ventricles T1**	751 ± 97.3	0.13				
**MANUAL SEGMENTATION**						
	O1		O2			
	**Mean ± St. Dev (mm** ^**3**^ **)**	**CV%**	**Mean ± St. Dev (mm** ^**3**^ **)**	**CV%**	**p value**	**DICE index**
**Caudate T2 L**	576.09 ±56.82	0.09	600.36 ±71.69	0.11	0.38	0.83
**Caudate T2 R**	586.77 ± 58.56	0.10	595.72 ± 64.26	0.10	0.31	0.83
**Hippocampus T2 L**	588.06±54.16	0.09	635.3 ± 62.15	0.09	0.07	0.69
**Hippocampus T2 R**	588.61±35.37	0.06	620.04 ±48.87	0.07	0.10	0.69
**Sub Nigra T2 L**	48± 11.04	0.22	50 ±9.73	0.19	0.64	0.79
**Sub Nigra T2 R**	54.19 ±9.73	0.17	57.9 ±9.90	0.17	0.37	0.76
**Lat Geniculate T2 L**	63.55 ± 4.78	0.07	71.2 ±14.83	0.20	0.11	0.72
**Lat Geniculate T2 R**	67.26 ± 6.92	0.10	74.68 ±11.98	0.16	0.09	0.73
**Med Geniculate T2 L**	78.19 ± 12.85	0.16	88.64 ± 18.22	0.20	0.13	0.71
**Med Geniculate T2 R**	85.50 ± 12.45	0.14	93.24 ± 14.27	0.15	0.19	0.73
**Putamen T2 L**	76.14 ± 11.88	0.15	77.25 ±13.46	0.17	0.84	0.74
**Putamen T2 R**	69.96 ± 9.50	0.13	71.65 ± 13.93	0.19	0.74	0.72
**Globus Pallidus T2 L**	55.06 ± 9.10	0.16	60.10 ± 11.36	0.18	0.25	0.72
**Globus Pallidus T2 R**	58.02 ± 8.96	0.15	61.79 ± 11.32	0.18	0.39	0.74

**Fig 1:**
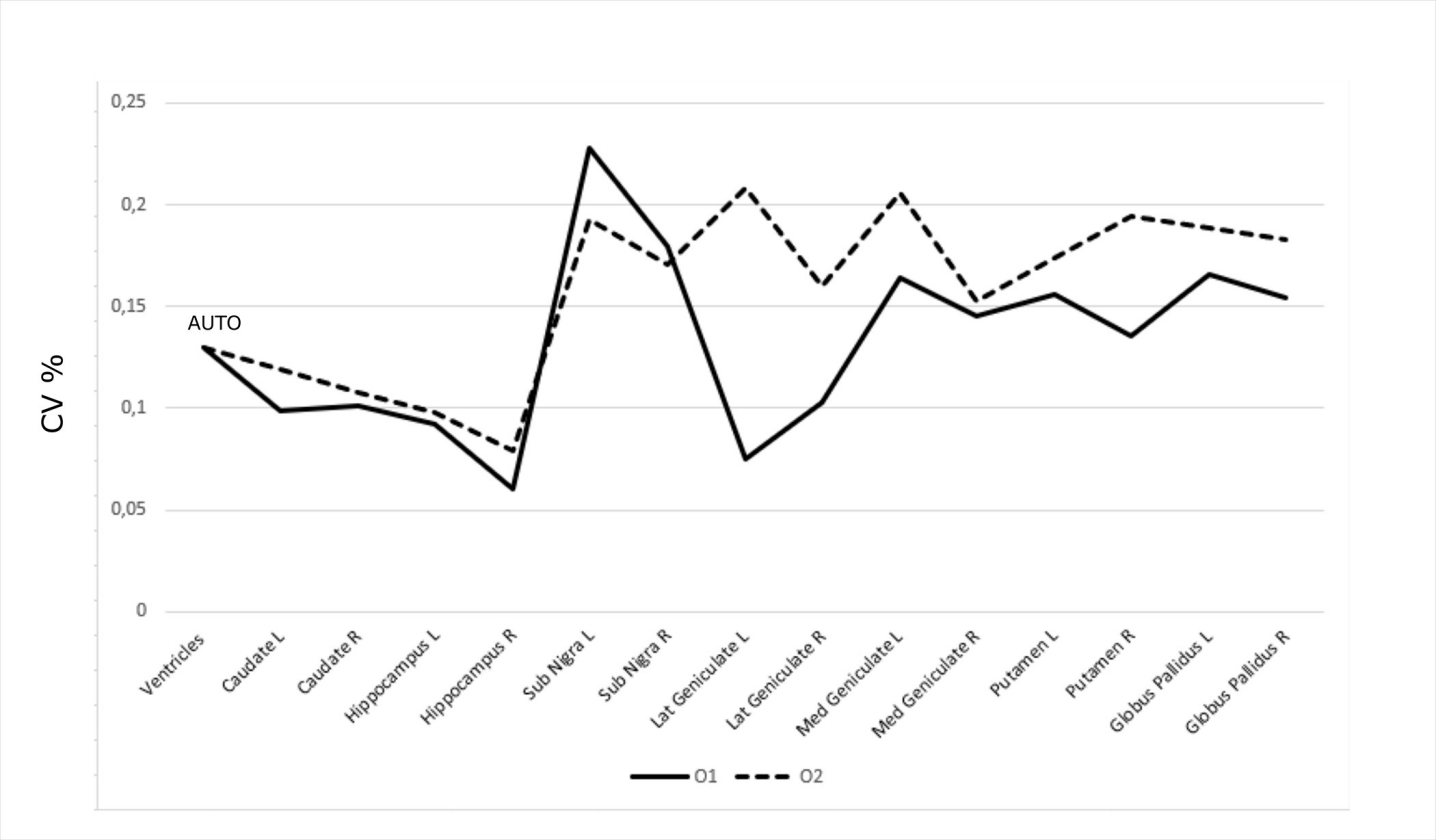
Segmentation stability across operators. The percentage variability of each structure segmented by operator O1 (solid line) and O2 (dotted line). It can be noted that the lowest variability was observed for both operators for the largest and more defined brain structures, thus suggesting that the MRI volume of the segmented structure is influenced by the actual size and its intrinsic contrast with the surrounding parenchyma. The ventricles have been segmented through an automatic approach (one segmentation—no variability).

For each operator (O1/O2), we report the mean and standard deviations of the volumes and their percentage variations expressed as CV. For all the considered structures, we tested the automatic segmentation described in [14,24]. However, as discussed below, we obtained reliable results only for the ventricles. Therefore, for this structure, we will refer to the automatic segmentation in the whole study. In the considered sample, we obtained a higher accuracy for the segmentation (see S1 Fig). The mean value of the volume was 751 mm^3^ (as compared to 818 mm^3^ as the mean volume from manual segmentations) and the standard deviation was 97.3 mm^3^ (as compared to 218 mm^3^ as the average standard deviation from manual segmentations). This shows that this estimate is more stable than the manual one, leading to a percentage decrease of the standard deviation of 55%. The values obtained from the automatic segmentation led to a CV = 0.16. For the other subcortical structures, due to the changes of contrast in T1 and T2, the automatic segmentation did not perform well so we relied on the manual approach. The Substantia Nigra and Geniculate showed the highest variability (around 20%) while the remaining structures showed variability of about 15% or less (see Fig 1).

To assess the agreement of the segmentations among the operators, the Dice Similarity index [25] was computed for each subcortical structure. As can be seen in Table 1, the results are encouraging showing that on average the Dice index was 0.75.

**Fig 2:**
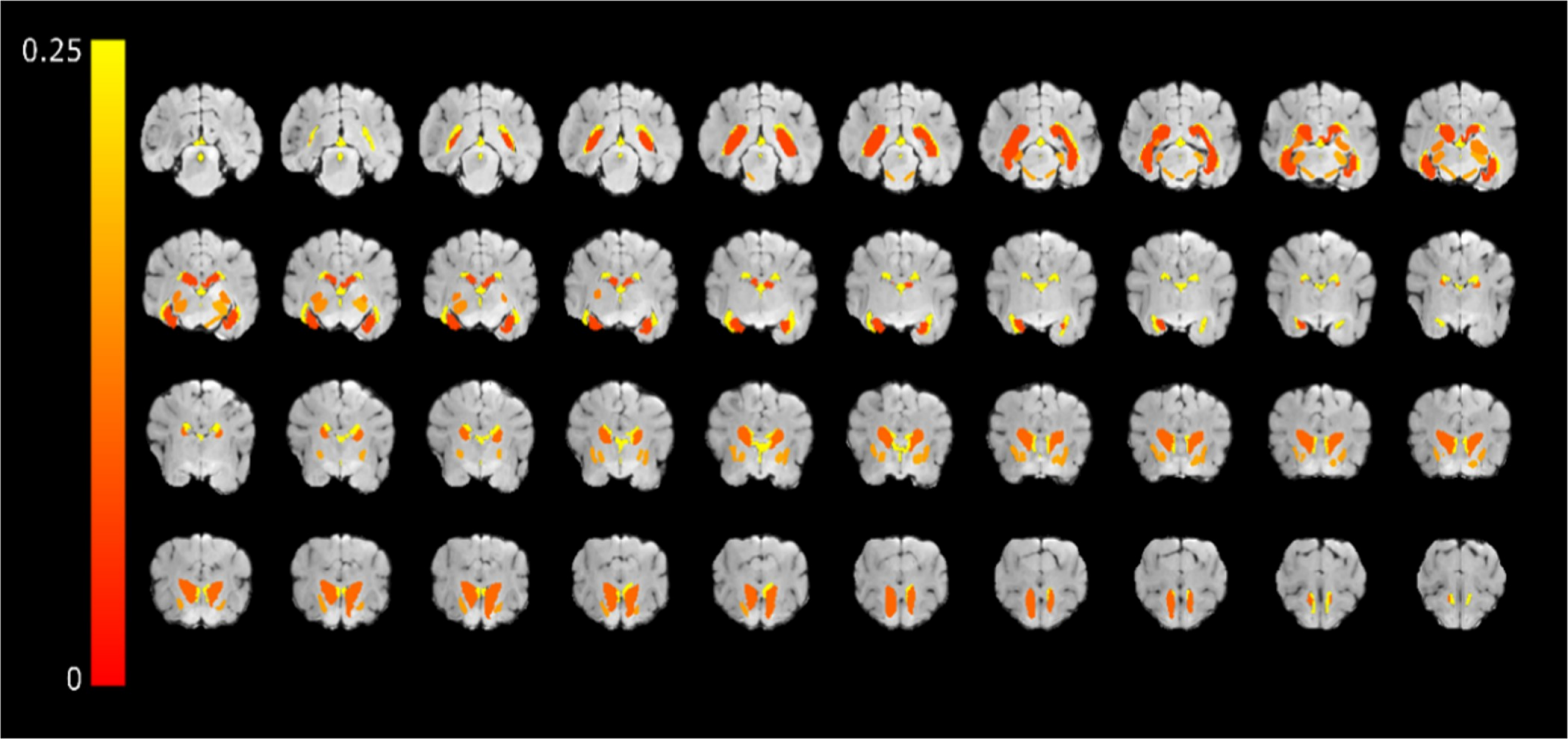
The spatial topography of CV of the considered structures. CV averaged across operators overlaid on T1w images of a representative dog.

The spatial topography of these variations is shown in Fig 2, where CV, averaged across operators, is overlaid on a T1w image of a representative dog.

**Fig 3:**
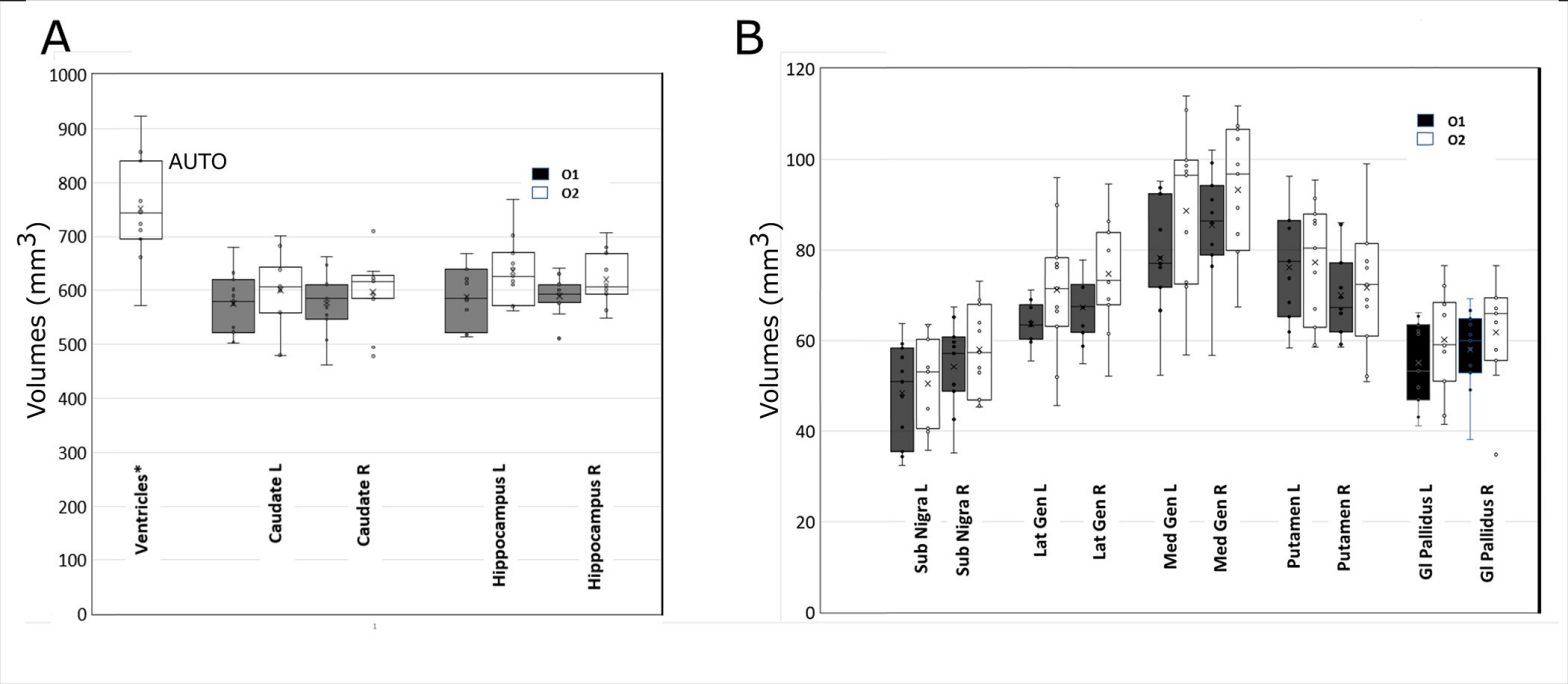
Data distribution of the segmented structures. Whisker plot for the distribution of the segmented volumes from the two operators O1 (black) and O2 (white). A) The set Ventricles, Caudate, and Hippocampus. Ventricles have been automatically segmented. No statistical differences have been observed. B) The set Substantia Nigra, Lateral and Medial Geniculate, Putamen and Globus Pallidus. In this set, no statistical difference was observed.

As can be seen in Table 1 and Fig 3, where the whisker plots of the analyzed structures are reported, a t-test showed no statistically significant differences between the two operators.

This seems to suggest that the segmentation of the considered structures is quite stable and reproducible across operators. Of note, for the Ventricles, we observed large variations also across operators when the manual segmentation was employed. This might be ascribed to the difficulty in manually identifying their border with cerebrospinal fluid (CSF). Also in this case the automatic segmentation outperformed the manual one. See S1 Fig where the two approaches are compared for a representative subject.

### A longitudinal study on brain structures

**Fig 4:**
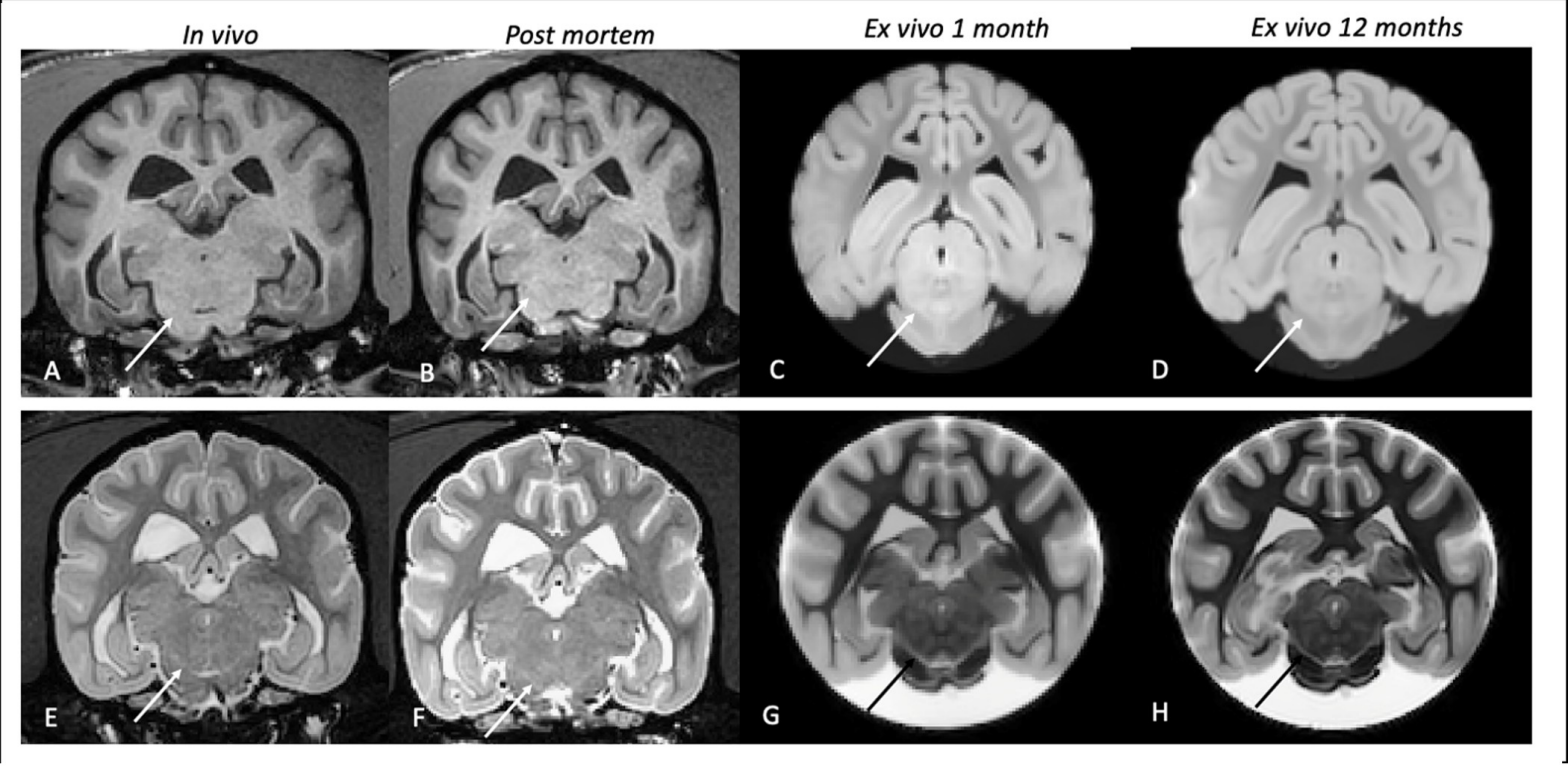
T1 and T2 progressive change of contrast. Panels A-D: T1w transverse section of the studied dog in the four different phases of the experiment, respectively in vivo (A), post mortem (B), ex vivo 1 month (C), and ex vivo 12 months (D). In vivo and post mortem grey and the white matter appeared respectively hypointense and hyperintense, on ex vivo images the contrast appeared exactly the opposite, with grey and white matter respectively hyperintense and hypointense. The solid arrows point at the Substantia Nigra and a progressive increase in contrast can be seen. Panels E-H represent the same four phases from T2w images. Here, rather than an inversion of the normal contrast, a sharp decrease in white matter intensity was observed in the ex-vivo phase. This leads to an increased definition of the contours of the different structures.

In this part of the study, for a representative dog, we assessed the longitudinal changes of segmentations performed *in vivo*, *post mortem*, and *ex vivo_1* (after one month in formalin) *and ex vivo_12* (after 12 months in formalin). First, we observed that both T1w and T2w images showed a change in signal contrast between the *post mortem* and *ex-vivo* data, see Fig 4.

**Fig 5:**
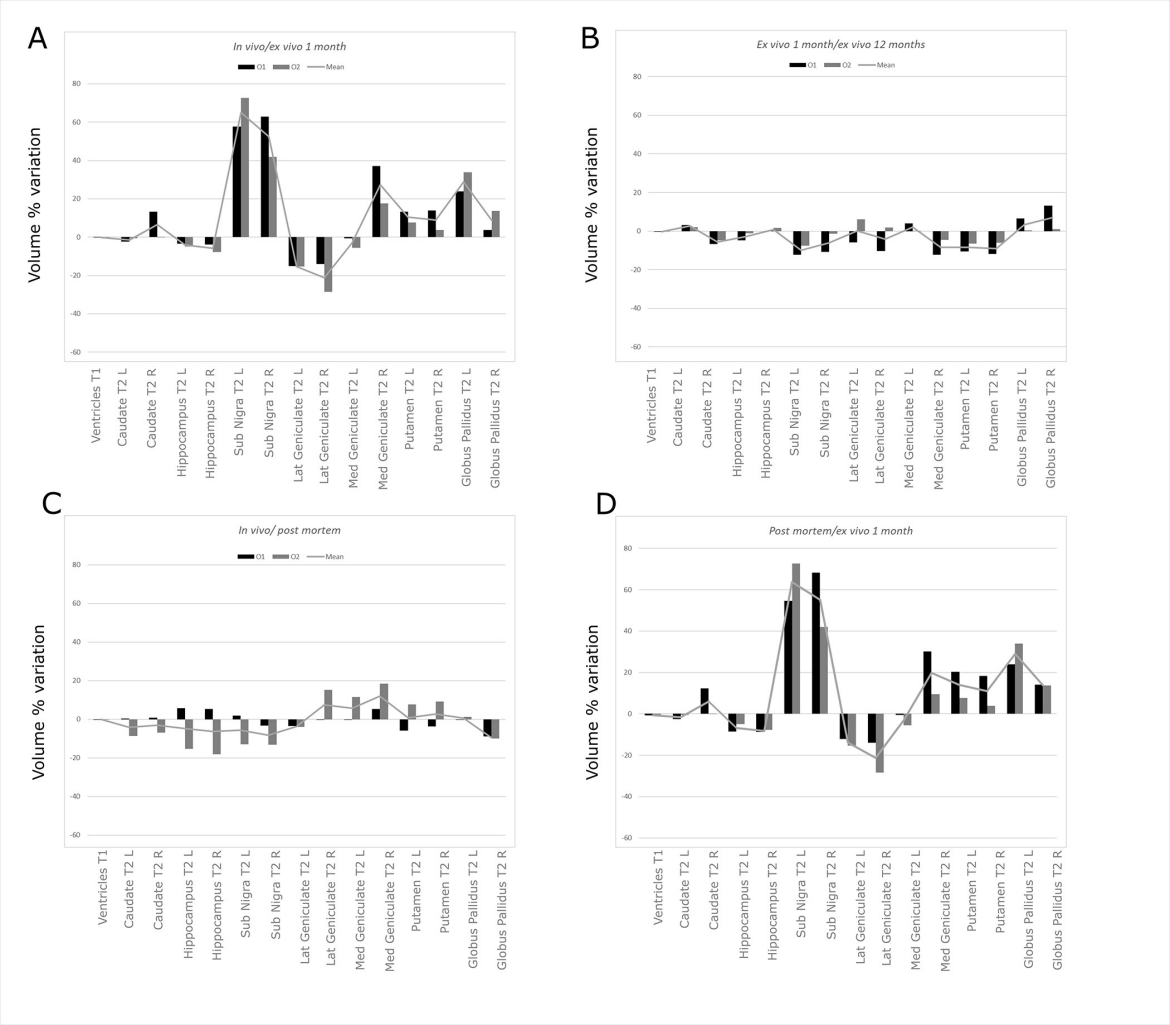
Longitudinal variations. The results for the ventricles refer to the automatic segmentation. A) Volume variation between the in vivo and post mortem phases. Volume variations were obtained for O1 (black), O2 (gray), and the mean trend (line). It can be observed that the volume variation is always lower than 20%. The trend is variable between the two operators. This aspect may be justified by the difficulty of clearly distinguishing the exact borders of the structures in-vivo. B) Volume variation between the post mortem and ex_vivo_1 (after 1 month) phases. Volume variations were obtained on each structure for O1 (black), O2 (gray), and the mean trend (line). It can be noted that the agreement between the two operators is higher than in the previous phase, with most structures experiencing an increase in volume from the post mortem to ex vivo phase. This is particularly interesting since the shift of the contrast after the time in formalin qualitatively seems to improve the visualization of the smallest structures. C) Volume variation between the in vivo and ex_vivo_1 phases. It can be noted that also in this case the operators agree and a trend similar to the previous figure is observed across the structures, i.e. most of the smallest structures appeared more clearly identifiable, thus justifying an increase in the volume. D) Volume variation between the ex_vivo_1 and ex_vivo_12 phases. It can be easily observed that the volume variation is relatively low between the two phases, with an overall agreement from both operators, thus suggesting that the time spent in formalin does not significantly influence the volume variation.

Specifically, on T1w images, while *in vivo* (A) and *post mortem* (B) grey and white matter (GM and WM) appeared respectively hypointense and hyperintense, on *ex vivo* images (C-D) the contrast seems to be the opposite, with GM and WM hyperintense and hypointense. This is evident in the Substantia Nigra, (white arrow in the figure). It can be observed that while in panels A) and B) this structure is barely visible, in panels C) (*ex vivo_1*) and D) (*ex vivo_12*) the contrast increases, and the borders of the structure are more prominent. On T2w images, it is observed a strong decrease in the white matter in both *ex vivo* phases, leading to an increased definition of the contours of the various structures. This is even more noticeable for the smaller structures (e.g. Putamen or Globus Pallidus), whose borders were difficult to define by both operators *in vivo* and *post mortem* phases (Fig 4E–4H). The volumetric percentage variations (see Materials and Methods) across time of the considered structures are reported in Fig 5. As in the previous analyses, the Ventricles were segmented automatically.

**Fig 6:**
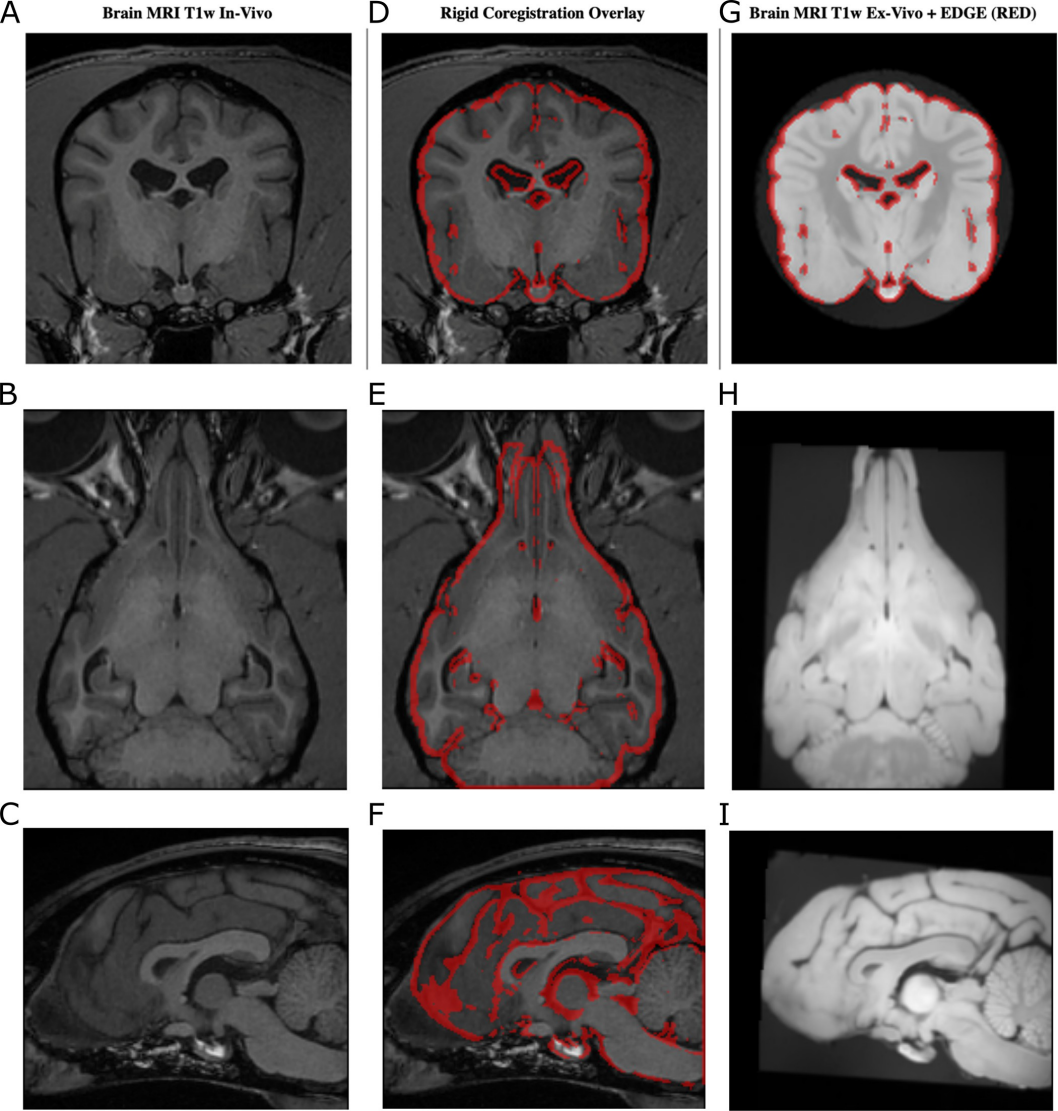
Data coregistration. T1w in vivo data are used as the reference to coregister the ex-vivo data with a 6-parameter coregistration approach (no scaling included). As an example, a representative subject is reported (A-C). The in vivo brain contours are overlaid to the ex-vivo data after the coregistration (D-F). It can be noted a good agreement. Analogously, the in-vivo brain contours overlaid on the ex vivo 12 months show that the rigid coregistration successfully aligned the data (G-I). Since no scaling was involved in the data transformation, this suggests that the brain did not experience any significant inflation/shrinkage.

As far as it regards the across-operator variability of the segmentations, also in this case, they seem reproducible in the various phases. The percentage variation of the volumes globally follows a similar trend for the two operators. Specifically, for the *in vivo/ post mortem* comparison (Fig 5A), the volumetric variation observed in the various structures is close to 12%, fluctuating at most around 20%, and can be thus considered low. Notably, in some structures such as the Lateral and Medial Geniculate nucleus, the volume increased by 18–20%. In this comparison (*in vivo* vs *post mortem*), especially for the *in vivo* images, both operators experienced some difficulties to delineate the borders of the investigated structures. They reported that this was independent of the structures’ dimension, i.e. it applied also to the largest structures. Although this holds for the *in vivo* phase, in the *post mortem/ex vivo* comparison, most structures showed a higher contrast. This led to more reproducible segmentations between operators, with the greatest variability of approximately 10% (Fig 5B–5D). However, we also observed for some structures a large increase in volume. This was not expected since the formalin fixations are reported to lead to shrinking and reduced tissue volumes [24]. This effect was particularly evident for Substantia Nigra (average increase of around 70% Fig 5B and 5C), followed by Globus Pallidus (around 30%) and Geniculate (around 20%). In general, for both *ex vivo* phases, the operators described an increased contrast in the borders of the various structures. The smallest structures were described to be sensitively easier to segment. Considering the previous findings, this suggests that the increase of contrast seems to be ascribed to the *ex-vivo* condition and thus the effect of fixation. Now, to study if this change remained stable over time, we compared the two *ex vivo* conditions (*ex_vivo_1* and *ex_vivo_12)*. As it can be seen in Fig 5D, the highest changes fluctuate around 20%. This suggests that the increased time spent in formalin did not influence significantly the volumes of the various structures. Basically, in this period, the structures’ volumes remained stable. This is an important finding suggesting that the segmentation can be performed even after a significant amount of time after fixation. To understand if these volume changes were induced by an overall shrinkage or inflation of the brain, we performed the coregistration of the data acquired at the different time points. As an example, in Fig 6, we report the borders of the brain extracted (for a representative slice) from *in-vivo* data overlaid to the T1w images obtained *ex-vivo*.

It can be noted that a rigid co-registration with 6 parameters, thus excluding any scaling factor, successfully coregistered the data. The same applies to the coregistration of the *ex-vivo* data after 12 months, see Fig 6 (right panel). The fact that no scaling factor was needed to coregister the data seems to suggest that the brain did not experience any significant inflation/deflation over time. Therefore, the changes observed in the volumes were likely due to a change in the signal contrast.

So far, the two samples of dogs have been treated separately. However, an interesting point would be if the longitudinal changes observed on a single dog hold also for the sample of 11 dogs. To address this aspect, in a future study, we will perform the same longitudinal study on a sample of dogs. However, with the data available at this stage, we tried to assess if there were statistical differences between the volumes obtained from the single dog and the 11-dog sample. To this aim, we considered the distribution of the volumes, structure by structure, obtained from the sample. We tested if the volumes obtained from the single dog (after 12 months in formalin) belonged to the same distribution, i.e. if they were statistically different. As it can be seen in Table 2, where we report the 95% confidence intervals and the test outcomes, apart from Right Hippocampus and Right Substantia Nigra, all structures were not statistically different. Ventricles were segmented using automatic segmentation.

**Table 2:** Comparison between the measurements obtained on the group of 11 dogs after 12 months in formalin and the ones obtained on the single brain dog after 12 months in formalin. Mean values between the operators are reported for each structure for both the group of the 11 dogs and the single brain dog after 12 months in formalin. A t-test revealed that only Hippocampus right and Sub Nigra R a significant difference was obtained (marked as *) between the two distributions.

	Mean_11 dogs	CI_11 dogs	Mean_exvivo12 months	t test
**Ventricles T1**	751 AUTO	459–1042	779	
**Caudate T2 L**	588.22	479.48–690.26	498,13	
**Caudate T2 R**	586.75	472–681.36	496.75	
**Hippocampus T2 L**	611.68	516.1–728.84	530.83	
**Hippocampus T2 R**	611.95	533.14–690.48	524.04	*
**Sub Nigra T2 L**	49.43	33.2–63.7	37.88	
**Sub Nigra T2 R**	56.09	39.8–70.58	36.86	*
**Lat Geniculate T2 L**	67.37	49.38–92.34	74.06	
**Lat Geniculate T2 R**	70.97	53.84–89.58	68.00	
**Med Geniculate T2 L**	83.41	54.88–112.1	65.03	
**Med Geniculate T2 R**	89.37	63.14–109.16	70.11	
**Putamen T2 L**	76.70	58.3–95.64	67.76	
**Putamen T2 R**	70.81	51.62–912	69.14	
**Globus Pallidus T2 L**	57.62	41.34–73.86	56.49	
**Globus Pallidus T2 R**	59.90	36.72–72.48	57.63	

The volumes obtained from the single dog, after 12 months in formalin, seem to be consistent with the volumes obtained from the 11 dogs’ sample. The mast majority, namely 86% of the structures, considered for the longitudinal study, belonged to the same distribution of the 11 dogs. This suggests that the considerations on the longitudinal changes observed on a single dog might hold also for the larger sample. Although encouraging, this point needs to be validated with a larger sample in a future study.

## Discussion

This work has been conceived with a dual purpose. First, we assessed the feasibility of brain segmentations on selected structures in the brain kept in formalin for one year. We focused on the reproducibility of the segmentations as a function of the operator and their intrinsic variability within the sample. This study was performed on a homogeneous sample of 11 dogs. Then, one dog (not part of the previous sample) was used for the longitudinal evaluation of the effect of death and fixation on MRI. We obtained that after one year in formalin, the segmentations seemed reliable and mostly reproducible across operators. Of note, we observed that the time spent in formalin increased the contrast for some specific structures, such as the Substantia Nigra.

The impact of Beagle cerebral models on MRI translational studies is currently increasing, e.g. in neurological diseases [26,27]. Since these animals can spontaneously develop brain disorders similar to humans, they are might be more relevant than rodent models with induced diseases [28–31]. Another major advantage is to monitor the disease non-invasively with the same MRI scanners used in medical facilities. This facilitates the translation of imaging biomarkers from animals to human patients. For this reason, several groups are interested in characterizing brain structures in this model. To this aim [12,14–16], canine MRI-based atlases are being developed. However, at the current stage, these are either based on a heterogeneous group of dogs, i.e. a mixed breed sample [3], or on Beagle dogs only, but considering only WM, GM, and CSF, and not specific structures [15]. Further, while these atlases were obtained from alive dogs, in [12] a mixed-breed atlas was obtained by coregistering *in vivo* with *ex vivo* data. Compared to these works, in our study, for the first time to our knowledge, selected structures have been segmented in brains fixed with Phosphate buffered (neutral) 10% formalin. This allows assessing the reproducibility of the volumetric estimates of such structures. This aspect has not been previously evaluated. In general, the segmentation can be perfomed manually or automatically. In line with [15,21], where brain templates were built from manually segmented data, here we adopted a manual segmentation. This is characterized by longer analysis time as compared to an automatic approach [32] which instead might result more accurate, especially for *in vivo* data [14,24]. However, in formalin-fixed brains, to perform the automatic segmentation [14,24] might be challenging. We tested this approach on our structures. We observed that the formalin fixation changed the MR signals affecting the conventional segmentation tools (SPM + TPM). Specifically, the fixation induces an increase in T1 and a decrease in T2, making WM and GM very difficult to differentiate. These effects lead to a worsening of the global SNR of the image and models based on the Gaussian mixtures seem not to perform well in this context. In fact, this difference in signal between *in-vivo* and *ex-vivo* data leads to difficulties in estimating the non-rigid (intra-modality) transformation to be applied to the atlas. The approach developed in [2], is very valuable, but it has been optimized on a T2 template obtained from in-vivo data. Nevertheless, this approach resulted very useful for the ventricles. In fact, for this structure the automatic segmentation resulted more accurate and stable than the manual one. We observed a decrease of 55% of the standard deviation of the ventricular volume. This finding is particularly important in our study, since the ventricles, based on the manual segmentation, resulted the most variable structure in terms of volumetric changes. This comparison with the automatic approach suggests that the manual one was questionable for this structure. However, apart from the ventricles, our findings suggest that the manual segmentation on this kind of data can be considered overall reproducible since only slight statistical differences were detected.

We observed that some structures were characterized by higher volume variations than others. This may be due to an intrinsic individual variability. This aspect is of interest since these structures may be targeted for specific experiments. Determining that there may be intrinsic variability could be crucial, for example, to distinguish a pathology- or drug-induced effect from a physiological variation. Since the volumetric estimation can assist in monitoring the progression of brain diseases, the reproducibility of MRI segmentation are important also in the clinical field [33].

Compared to histopathological analyses, the MRI-based segmentation of formalin-fixed brains has several advantages. First, structural abnormalities can be assessed within the entire brain without altering the original structures. Second, the fixation allows to analyze the data multiple times in any plane [19,34], preserving the structure of the tissues. In fact, after death, the brain undergoes microbial degradation, autolysis, and breakdown of cell membranes. The chemical fixation tends to preserve the macromolecular structure, providing the longitudinal stability required for extensive scanning times. Nevertheless, a series of artifacts and changes are expected. It is known that the formalin fixation may alter the relaxation times. This is due to the induced tissue dehydration, crosslinking, and reduced transmembrane water exchange [34] that lead to a T1/T2 shortening. This results in a higher spatial resolution in terms of borders visualization, for technical details see [19,35]. This is in line with what we observed in the *ex vivo* phases. Both operators found an increase in the volumes, especially for the smallest structures, that were judged easier to segment. To support this interpretation, we carefully checked the brain volumes by co-registering the data across the three phases. We obtained no significant changes. Therefore, this apparent increase in volume did not correspond to a real increase in the size of the structure but to an increase in contrast in the structures’ borders, allowing a more accurate segmentation. Evidently, before fixation, the structures’ volumes were under-estimated due to low contrast. This is in line with previous findings, see for example [36] where it was reported that the qualitative image evaluation significantly improved after fixation. The structure segmentations were described to be easier than *in vivo* images.

Another effect of formalin fixation, reported in the literature, is the tissue shrinkage which may be inhomogeneous among the various brain structures, see [21]. For example, it has been observed that ventricles may experience filling or emptying according to pressures from the surrounding tissue. The shrinkage of surrounding tissue may not always be paired with an expansion of the ventricles [37] with those tissues experiencing a “positive formalin effect”. This is characterized by a swelling effect caused by the osmotic pressure of the formalin solution [38].

Another important aspect is the type of fixation. Typically, with an immersion fixation we expect larger brain volumetric differences to occur than with perfusion. When the fixative agent penetrates the tissues, it ensures a better fixation. During sampling and removal from the skull, it maintains the original geometry of soft tissues, by limiting distortion. Since a stable flow rate of fixative is fundamental, in our case, this was ensured by means of a peristaltic pump. This allowed relatively rapid perfusion. We used a flow rate of 15mL/min, as in the protocol described in [39]. In [20] the authors emphasize the importance of perfusion flow rate, the form of perfusates and observed some fixation artifacts. They noted a hyperintense rim around the brain using perfusates reconstituted from powder and delivered at a high flow rate. With our protocol, we did not observe any hyperintense rim around the brain. Furthermore, our technique minimizes exposure to chemical substances such as formaldehyde (inherent toxicity, eye irritation) increasing operators’ safety.

In the adopted protocol, how often the formalin is renewed in the solution, plays a fundamental role. High concentrations of methanol (included as a stabilizing agent in commercial formalin solutions) can induce artifacts in the neuropil. This happens when the fixation time of the samples is prolonged in unrefreshed formalin. This has been observed both at high (9.4 T) [40] and low field MRI [41] in specimens destined for fine histological morphometry, immunohistochemistry, or ultrastructural analysis. Several works suggest long stability of the tissues in formalin of around a few months (2–3 months), see for example [42]. Based on this literature, we have been conservative and replaced the formalin every six weeks.

In post mortem MRI studies, to work with the brain inside the skull, is beneficial for the preservation of the structures. The presence of the skull limits both structural change (particularly expansion) and exposure to the surrounding medium. However, we are aware that minimal deformation may result in the end, see [43].

The age might influence the process of formalin fixation due to possible parenchymal atrophy observed in senile patients [47]. Nevertheless, no specific corrections can be adopted to account for the fixative effects related to age or body weight. Of note, in this study, the subjects were uniform in weight and age. However, we acknowledge that these aspects have not been investigated at this stage and represent an interesting topic for future studies.

Finally, from an histopathological point of view, the formalin per se has no species-specific effect. The difference lies mainly in the different brain sizes. In the literature, several papers have been published on perfusion fixation in various laboratory species but this is scarce in dogs, see [48]. The results are divergent, and they change according to the dilution percentage [49] and the type of formalin used [50]. A fixation protocol for dogs is yet to be standardized.

Taking into account all these considerations, our findings suggest that the amount of time (12 months) spent in formalin seems not to influence the volumes of the structures: their percentage variation did not exceed 10%. Of note, the volumes estimated for some of these subcortical structures, such as the Ventricles, Caudate Nucleus, and Hippocampus, are in line with previous works on Beagles (non in formalin), see [14]. These estimates are in agreement considering the reported confidence intervals.

The observed stability for the brain in formalin, suggests that the same brain can be potentially used for several studies, even after some time, without the risk of significant structural changes. This observation is in line with previous studies reporting that the brain structures remain relatively stable for 6 months post-mortem [44]. This applies also to human medicine where it has been shown that fixation leads to no significant leaching of iron in long-term storage [45]. Further, WM components, including myelin, seem to be well preserved [46].

This work paves the way for future studies. To rely on reference volumes might be particularly interesting when MRI is compared to histopathology or to investigate disorders causing volumetric modifications, e.g. in the case of epileptic dogs [47]. A thorough understanding of the anatomical features of the brain in clinically normal dogs is essential. This is a prerequisite to accurately interpret any pathological changes seen in MRI. It may also be a useful tool for diagnosing neuropathology in dogs retrospectively, i.e. studying brains fixed for some time [48]. In fact, post mortem imaging allows correlating in vivo MRI results with any histological sections. Probabilistic maps can be obtained based on ex vivo MRI data. These, after being validated on histology, can be translated into an in vivo model [49]. These ex-vivo/in-vivo mapping is promising in many applications such as brain biopsy [50] and stereotactic radiotherapy [51]. Further, this approach provides archival and replicable images of canine brain samples prior to permanent sectioning. These images can complement evidence obtained from both traditional neuropathological observations and in vivo neuroimaging. This may identify histological, cellular, and molecular mechanisms of structural damage preexisting in genetically disposed groups, see [34].

To summarize, based on our findings, *post mortem* MRI based segmentation seems to be a useful and accurate tool that allows longitudinal studies. However, especially for the observed longitudinal variations, these findings need to be further validated on a larger sample.

## Supporting information

S1 Fig Comparison manual vs automatic segmentation of the ventricles. A,C) The automatic segmentation of the ventricles (red) produced more accurate borders of these structures as compared to the manual segmentation, reported in B), D). Some parts were missing in the manual segmentation (see yellow box), some borders were less accurate as well as some internal structures resulted inhomogeneous (yellow arrows).
(TIF)
