# Supplementary figures and images for: Volumetric assessment and longitudinal changes of subcortical structures in formalinized Beagle brains
Source: PLoS One. 2022 Oct 7;17(10):e0261484. doi: 10.1371/journal.pone.0261484 (PMC9543981; doi:10.1371/journal.pone.0261484)

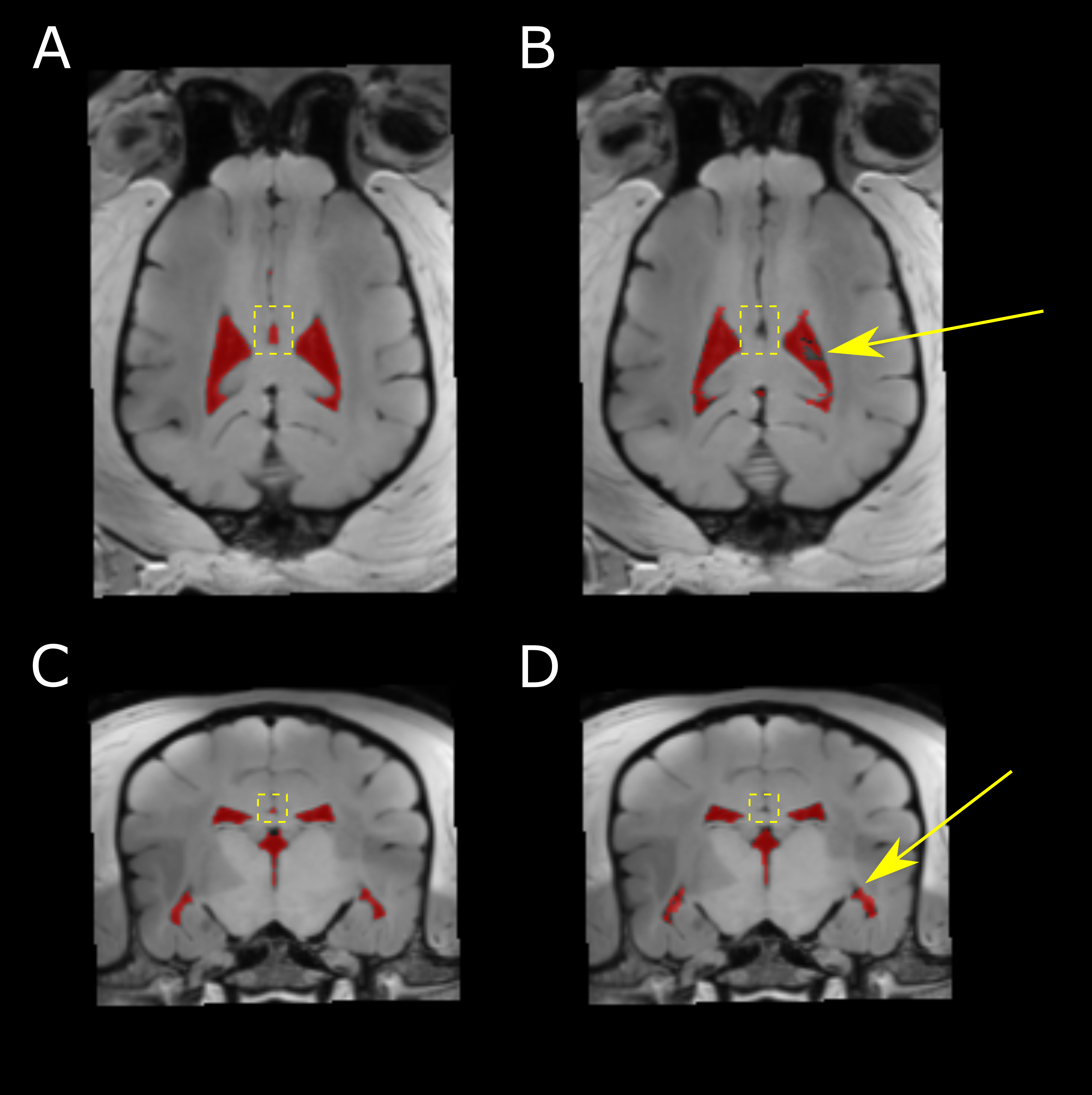

Supplement: S1 Fig — A,C) The automatic segmentation of the ventricles (red) produced more accurate borders of these structures as compared to the manual segmentation, reported in B), D). Some parts were missing in the manual segmentation (see yellow box), some borders were less accurate as well as some internal structures resulted inhomogeneous (yellow arrows). (TIF) [file pone.0261484.s001.tif]
